# Outcomes of surgical vs medical treatment for cholecystitis among people living with dementia

**DOI:** 10.1002/alz.087058

**Published:** 2025-01-09

**Authors:** Rachel R Adler, Lingwei Xiang, Samir K Shah, Clancy J Clark, Dae Kim, John Hsu, Josh Lin, Stuart Lipsitz, Susan Mitchell, Joel S Weissman, Emily Finlayson

**Affiliations:** ^1^ Brigham and Women’s Hospital, Boston, MA USA; ^2^ University of Florida, Gainesville, FL USA; ^3^ Wake Forest School of Medicine, Winston‐Salem, NC USA; ^4^ Hebrew SeniorLife Marcus Institute for Aging Research, Boston, MA USA; ^5^ Massachusetts General Hospital, Boston, MA USA; ^6^ Hebrew Senior Life’s Hinda and Arthur Marcus Institute for Aging Research, Boston, MA USA; ^7^ University of California ‐ San Francisco, San Francisco, CA USA

## Abstract

**Background:**

Cholecystectomy is considered the definitive treatment option for cholecystitis, and patients living with Alzheimer’s Disease and Related Dementias (PLWDs) are at risk for increased mortality, complications, and delirium. However, the effect of different treatment options for cholecystitis among PLWDs has not been elucidated; therefore, this study compares outcomes following cholecystectomy, cholecystostomy tube, and medical management of cholecystitis among this high‐risk group.

**Method:**

We conducted a retrospective analysis of Medicare claims data from 1/1/2016‐12/31/2020. The cohort comprised Medicare PLWDs with continuous fee‐for‐service coverage who were age 66+ and were admitted to an acute care facility with a primary diagnosis of cholecystitis between January 1, 2017 and December 31, 2018 and who had no admissions for cholecystitis in the year prior. We used inverse propensity weighting regression to adjust for confounding by indication. We compared mortality, length of stay, intensive interventions (i.e., intubation), discharge to a higher level of care, and readmissions among patients treated with cholecystectomy, cholecystostomy tube, and medically managed.

**Result:**

We identified 10,187 individuals who met inclusion criteria. Of these, 5,888 (58%) were treated with cholecystectomy, 1,373 (13%) with cholecystostomy tube, and 2,931 (29%) were managed medically. After propensity weighting, we found PLWDs undergoing cholecystectomy had lower mortality (HR 0.66, p<.001), greater risk of delirium (OR 1.26, p = .001), intensive interventions (OR 1.67, P<.001) and discharge to a higher level of care (OR 1.15, p = .007), but lower risk of readmission (HR 0.88, p<.001) compared with subjects managed medically. Likewise, subjects with cholecystostomy tube had greater risk of delirium (OR 1.75, p<.001) and discharge to a higher level of care (OR 1.80, p<.001), but no differences in mortality, intensive interventions or readmissions compared to those managed medically.

**Conclusion:**

Over half of PLWDs experiencing acute cholecystitis received definitive surgical treatment during the index admission. Cholecystectomy in this population was associated with reduced mortality compared to non‐surgical management, but no improvement in other outcomes such as delirium and discharge to a higher level of care. These data can help inform decision‐making discussions for patients with ADRD and their care partners when considering treatment options for acute cholecystitis.

